# Supplementary material for: Enhanced Diabetic Wound Healing Using Electrospun Biocompatible PLGA-Based Saxagliptin Fibrous Membranes
Source: Nanomaterials (Basel). 2022 Oct 25;12(21):3740. doi: 10.3390/nano12213740 (PMC9659155; doi:10.3390/nano12213740)
Supplement: Supplementary file 1 [file nanomaterials-12-03740-s001.zip › nanomaterials-1942964-supplementary.pdf]

Enhanced Diabetic Wound Healing Using Electrospun Biocompatible PLGA-Based Saxagliptin Fibrous Membranes

Chen-Hung Lee [1,*], Shu-Chun Huang [2,3,4], Kuo-Chun Hung [1], Chia-Jung Cho [5,*] and Shih-Jung Liu [6,7,*]

[1] Division of Cardiology, Department of Internal Medicine, Chang Gung Memorial Hospital-Linkou, Chang Gung University College of Medicine, Taoyuan 33305, Taiwan
[2] Department of Physical Medicine and Rehabilitation, New Taipei Municipal Tucheng Hospital, New Taipei City 23652, Taiwan
[3] Department of Physical Medicine & Rehabilitation, Chang Gung Memorial Hospital, Taoyuan 33305, Taiwan
[4] College of Medicine, Chang Gung University, Kwei-Shan, Taoyuan 33302, Taiwan
[5] Institute of Biotechnology and Chemical Engineering, I-Shou University, Kaohsiung 84001, Taiwan
[6] Department of Orthopedic Surgery, Bone and Joint Research Center, Chang Gung Memorial
Hospital-Linkou, Taoyuan 33305, Taiwan
[7] Department of Mechanical Engineering, Chang Gung University, Taoyuan 33302, Taiwan
* Correspondence: chl5265@gmail.com (C.-H.L.); ppaul288@isu.edu.tw (C.-J.C.); shihjung@mail.cgu.edu.tw (S.-J.L.); Tel.: +886-3-2118166 (S.-J.L.); Fax: +886-3-2118558 (S.-J.L.)

Supplement 1: The percentage of the water content

Table S1. The percentage of the water content in 24 hours

Time (h)	Saxagliptin/PLGA group	Pristine PLGA group	*p* value
0.5	146 ± 8	57 ± 6	< 0.001
1	138 ± 25	55 ± 10	< 0.001
2	161 ± 27	42 ± 35	< 0.001
3	147 ± 9	21 ± 4	< 0.001
8	163 ± 30	32 ± 12	< 0.001
24	193 ± 14	40 ± 7	< 0.001
